# Physical Activity and Overnutrition Among 8–11-Year-Old School Children in Thika Town, Kenya

**DOI:** 10.1155/jnme/1537082

**Published:** 2025-05-29

**Authors:** Margaret Wanjiru Mburu, Peninah Kinya Masibo, Anselimo Makokha, Shehu Shagari Awandu, Patrick Opiyo Owili

**Affiliations:** ^1^Department of Community Health, Amref International University, Nairobi, Kenya; ^2^Global Programs for Research and Training Affiliate of the University of California San Francisco (UCSF), San Francisco, California, USA; ^3^School of Food and Nutrition Sciences, Jomo Kenyatta University of Agriculture and Technology, Nairobi, Kenya; ^4^Department of Biological Sciences, Jaramogi Oginga Odinga University of Science and Technology (JOOUST), Bondo, Kenya; ^5^Department of Research and Related Capacity Strengthening, African Population and Health Research Centre (APHRC), Nairobi, Kenya

**Keywords:** Kenya, obesity, overnutrition, overweight, physical activity, school children, undernutrition, urban settings

## Abstract

**Background:** Childhood overnutrition is a growing public health concern in the 21st century. It is a risk factor for adult obesity and noncommunicable diseases. If no action is taken, it is estimated that 208 million boys and 175 million girls aged 5–19 years will be living with obesity by 2035. This study aimed to determine the physical activity status and prevalence of overnutrition among school-going children aged 8–11 years and further explored the associations.

**Methods:** A cross-sectional study design was used among school-going children 8–11 years of age in Thika town, Kiambu County, Kenya. A total of 281 children were sampled. The physical activity levels were assessed using the validated physical activity questionnaire for older children. The BMI for age Z-scores (BAZ) was calculated based on weight and height measurements, and children with a BAZ score of +1 standard deviation were considered to have overnutrition. Data were analyzed using univariate, bivariate, and multivariate techniques. Logistic regression was employed to determine associations between the independent variables and the primary outcome.

**Results:** The prevalence of overnutrition among the children was 11%. On the other hand, 22.4% of the children were underweight. Most of the children (86.5%) attended PE classes. More than half (54.8%) of the children were physically active. Physical inactivity and attending PE classes were not associated with overnutrition. Overnutrition was significantly higher among private than public school children (aOR 2.641; 95% CI = 1.013–6.887, *p*=0.0047).

**Conclusion:** There is a presence of overnutrition in school children in Thika town, Kenya. The same population is also undernourished, thus demonstrating the double burden of malnutrition. Almost half of the children were physically inactive. An integrated approach to early detection, prevention, and management of malnutrition in children aged 5–19 years is needed. These findings have implications for public health interventions in preventing childhood obesity. Interventions could prioritize encouraging physical activity through school-based education, improvement of community infrastructure, and policy approaches. Multisectoral collaboration can create solutions that encourage active commutes with general obesity prevention.

## 1. Background Information

Overnutrition, marked by overweight and obesity, poses a significant and multifaceted public health crisis that impacts populations worldwide. Once considered a concern for high-income countries, it is increasingly becoming a problem in sub-Saharan Africa (SSA). Recent studies conducted across various SSA countries suggest an upward trajectory in childhood overweight and obesity [1]. Additionally, none of the countries has successfully reversed the trends of obesity [2]. It manifests in a disproportionate build-up of body fat that poses a risk to poor health outcomes. Morbidities associated with overnutrition are the fifth leading cause of mortality worldwide [3]. In 2018, the obesity society categorized obesity as a disease due to the numerous biological changes caused by obesity [4].

Middle childhood, between 6 and 11 years, is an important developmental stage since the foundations for health and well-being are being set [5]. Children in this stage are usually enrolled in primary school, which provides opportunities to develop their foundational skills for higher education and economic, interpersonal, social, and civic responsibilities later in life [6]. According to the Global Nutrition Report (2021), approximately 2.2 billion people are overweight globally, of which 772 million are obese. In children aged 5–9 years, the prevalence of overweight and obesity has increased from 17.0% among boys and 15.5% among girls in 2010 to 24.5% (boys) and 21.4% (girls), respectively, in 2019. An increase has also been observed among adolescents aged 10–19 years, from 14.4% and 13.8% in 2010 to 20.2% (male) and 18.4% (female), respectively, in 2019. Studies conducted in urban Kenya (8–14 years) and Nepal (6–13 years) revealed a 21% and 25.7% prevalence of overnutrition, respectively [7, 8]. The Global Burden of Disease 2019 data approximate that 206 million children aged 5–19 years will be living with obesity by 2025 [9].

In children, overweight and obesity have been reported to have detrimental effects as they contribute to the onset of chronic noncommunicable diseases (NCDs), which were once thought of as adult problems. Such diseases include hypertension, dyslipidemia, type 2 diabetes, difficulty breathing, mental health problems, and joint problems [1, 9]. Overweight and obesity result from an imbalance between caloric intake and caloric utilization [3]. This is due to a complicated interaction between genetic, metabolic, and behavioral practices; cultural and environmental factors; life events; marketing; mental health; lack of sleep; and stigmatization [10–12].

To curb the rise in these challenges, the WHO recommends about an hour of moderate to vigorous intensity physical activity (MVPA) per day for school-aged children between 5 and 17 years as one of the low-cost and evidence-based interventions [13]. Despite the known benefits of regular physical activity in reducing adiposity by increasing energy expenditure, about 80% of children in schools globally do not meet the requirements [14]. Kenya is experiencing a physical activity transition, with urban children being more inactive than those from rural areas [15]. A study conducted in Nairobi showed that only 12.6% of children 9–11 years old were physically active [16]. A recent WHO report also revealed a significant prevalence of physical inactivity among children aged 11–17 years in Kenya, with 85% of males and 89% of females being inactive [17]. Children accruing an average of 1 h of MVPA daily have shown to have improved bone health, cardiorespiratory and muscular health and fitness, cognition, reduced adiposity, and decreased risk of depression [18, 19].

Childhood overnutrition is now recognized as a critical threat to global health in the 21st century, with an estimated 42 million children aged 5–19 years affected. The number of obese children in Kenya is on the rise, with more than one million children aged 5–19 years projected to be obese by the year 2030, driven by urbanization and lifestyle. For this reason, the Sustainable Development Goals (SDGs), specifically goal (2.2), propose ending all forms of malnutrition. Furthermore, there is a call for global nutrition targets to be extended to children aged 5–19 years to prevent the increase in obesity and diet-related NCDs and reduce insufficient physical activity by 10% [20].

Despite this, there is a scarcity of literature on evidence of the burden of childhood overnutrition from LMICs, and even fewer studies offer disaggregated, context-specific findings on physical activity and overnutrition among school-aged children [1, 21]. Most studies assessing the prevalence of overnutrition among Kenyan children have focused on broader age groups, urban regions such as Nairobi, or national-level estimates, leaving a gap in understanding the periurban contexts such as Thika. This study aimed to assess the levels of physical activity and occurrence of overnutrition among school-going children aged 8–11 years in Thika town, Kiambu County, Kenya. The findings contribute to the scant empirical work directly connecting physical activity habits and overnutrition in 8–11-year-olds. Middle childhood remains underrepresented in the research and developmental agendas despite being a critical window for early prevention. Therefore, this study's findings can potentially inform targeted policy and public health interventions at the county and community levels.

## 2. Methods

### 2.1. Study Design and Population

A descriptive and analytical quantitative cross-sectional study design was used to investigate the relationships between overnutrition, sociodemographic characteristics, and physical activity. The target population was school-going children 8–11 years old whose families resided in Thika town. This is an urban setting in Kiambu County, Kenya. Children were excluded if sick or injured during the data collection period or those with conditions that impede physical activity, such as those in a wheelchair or requiring assisted walking. Children whose home residence was not in the selected subcounty were also excluded. The place of residence was determined from the school records. The parents' consent and children's assent were obtained before data collection. Data collection was conducted in the second academic term of 2024, between May and July.

### 2.2. Sample Size Determination

The sample size was determined using the Open-Epi Version 3.01 (Open Epidemiological Statistics for Public Health) statistical program. Ministry of Education data provided by the education office in Thika town indicated that the pupil population education office was 41,793 as of July 2022. There is a 21% prevalence of overnutrition among children 10–12 years old in urban Kenya [22], with a 95% level of confidence, a margin of error of 5%, and a design effect of 1.0. This brought the estimated sample size to 253 participants. We adjusted the minimum sample size upward by 10% to account for nonresponse and increase statistical power, making the final sample size 281 participants.

The study employed multistage sampling. The sample sizes from private and public schools were stratified based on the population sizes for different types of schools. It was determined that the students who met the inclusion criteria based on age were students in Grades 4, 5, and 6. However, more students in Grades 4 and 5 met the criteria compared to Grade 6, where most students were aged 12 years and above. Once the number of schools and the number was determined, simple random sampling was employed to select the specific schools. The number of students from each school and each grade was determined using proportionate sampling techniques, given the difference in population sizes for private and public schools and the difference in age categories between the grades. Thus, the initial sample size considered 50% from Grade 4, 135 pupils; 40% from Grade 5, 108 pupils; and 5% from Grade 6, 13 pupils.

### 2.3. Data Collection

#### 2.3.1. Physical Activity

An interviewer-administered electronic-based questionnaire was used for data collection using the Kobo toolbox and Kobo Collect mobile application. Physical activity was assessed by adopting the Physical Activity Questionnaire for older children (PAQ-C), validated for use among children aged 8–14 years [23]. It consists of ten questions, each assessing different aspects of physical activity. It was completed by evaluating the children's frequency of participation in various activities in their spare/leisure time. We adopted activities that reflect the actual situation of a typical Kenyan child, which included jogging, walking for exercise, skipping rope, playing tag, “*bladder*” (involves securing an elastic rope and having one or two individuals leap while traversing the rope), “*kati*” (an iteration of dodgeball which involves avoiding the ball when thrown in your direction), table tennis, football, hop, step and jump, hide and seek, cycling, volleyball, handball, and netball. Data were collected on the perceived level of physical activity during physical education (PE) classes, activities during break time and on lunch breaks (other than eating), number of days in the past week they had engaged in physical activity in the evenings, and immediately after school, the number of times they engaged in physical activity on weekends and perception of the children's level of physical activity. The responses from these questions were used to derive a summary score by calculating the average composite score of the nine items assessed. Per the protocol, a PAQ-C score of 1 and 5 denotes low and high physical activity, respectively. The cutoff points of a score ≥ 2.75 were used to indicate over 60 min of MVPA [24]. Additionally, the pupils were asked about participating in PE sessions and walking to school.

#### 2.3.2. Anthropometric Measurements

Height and weight were assessed using a portable SECA stadiometer and SECA digital weighing scale to the nearest 0.1 cm and 0.1 kg, respectively. The weight and height measurements were taken per the National Health and Nutrition Examination Survey (NHANES) anthropometry procedure manual (revised January 2017) [25]. All measurements were conducted twice, and an average was calculated. The BMI for age and sex Z-scores (BAZ scores) were calculated using Epi info Version 3.5.4 and interpreted per the WHO reference standards for children 5–19 years. Children were classified as underweight, normal overweight, and obese if their BAZ scores were <−2 SD, >−2 SD to < 1 SD, >+1SD, and >+2 SD, respectively [26].

### 2.4. Data Management and Analysis

Data from the Kobo Collect toolbox were downloaded, cleaned, coded, and then analyzed using Statistical Packages for Social Sciences (SPSS) Version 25. Measures of central tendency, frequencies, and proportions were used to describe the continuous variables. Inferential statistical tests included Chi-square and Fisher's exact tests (for observations with less than 5 cell counts) to assess the association between childhood overnutrition and the independent variables. The multivariate logistic regression analysis was used to estimate odds ratios for factors that showed statistical association at the bivariate level. Statistical significance was determined at a 95% confidence interval with a *p* value < 0.05.

## 3. Results

### 3.1. Characteristics of School Children

The mean age of the school children was 9.88 (±0.05). Over half of the respondents were girls, 55% (*n* = 155). More than half, 52% (*n* = 146), of the respondents attended private schools. About half, 51.9% (*n* = 147), of the respondents were in Grade 5. The average weight of the school children was 32.12 (±7.52) kg, while the mean height was 141.55 (±7.87) cm. About one-fifth, 22.4% (*n* = 63), were underweight, 10.3% (*n* = 29) were overweight, and 0.7% (*n* = 2) were obese. The prevalence of overnutrition, combining overweight and obesity, was therefore 11% (*n* = 31), as shown in Table 1.

### 3.2. Physical Activity of the School Children

Of the 281 school children, 86.5% (*n* = 243) participated in PE while 13.5% (*n* = 38) did not. More than a third of the respondents (37.4%, *n* = 105) participated in PE on one day, while 12.8% (*n* = 36) participated in PE classes every day of the school week. Most of the respondents who had PE sessions, 79.8% (*n* = 194), reported having the sessions lasting about 35 min. The mean duration of the PE sessions recorded by the respondents was 33.92 (±13.03) minutes, while the mode and the median duration were 35 min. More than half of the children (65.5%) routinely walked from their homes to school, and 38% walked for over half an hour. The mean PA score was 2.85. More than half, 54.4% (*n* = 153), had an average score of 3, and only 0.4% (*n* = 1) of the respondents had a score of 5. More than half of the children, 54.8% (*n* = 154), were classified as active, as shown in Table 2.

### 3.3. Relationship Between the Children's Characteristics and Overnutrition

As shown in Table 3, overnutrition was significantly associated with school type (*p* = 0.003) and walking to school (*p* = 0.006) at the bivariate level.

The odds ratios were then estimated through multivariate logistic regression models. Overnutrition was significantly higher among school children aged 8–11 years in private schools compared to public schools (cOR 3.597; 95% CI = 1.496–8.652, *p* = 0.005). There was a significantly higher prevalence of overnutrition among children who did not walk to school than those who walked (cOR 2.997; 95% CI = 1.399–6.419, *p* = 0.004). After adjusting for the type of school, the odds ratios of overnutrition were still higher among those who did not walk to school. However, the difference was not statistically significant (aOR 2.017; 95% CI = 0.875–4.650, *p* = 0.1). This is shown in Table 4.

## 4. Discussion

Our findings revealed an 11% prevalence of overnutrition among school-going children in an urban area of Kenya. These results are consistent with studies conducted in urban settings in Cote d'Ivoire and Tanzania, which also showed averages of 10% overweight and less than 5% obesity [30, 31]. These studies underscore the growing prevalence of overnutrition, rapidly emerging as a significant public health issue in LMICs [32]. Since obesity in childhood affects all organ systems, it contributes to an increased risk of NCDs such as type 2 diabetes mellitus, hypertension, gastroesophageal reflux disease, obstructive sleep apnea, dyslipidemia, musculoskeletal complications, and negative psychological experiences. Furthermore, children who are obese have a higher likelihood of being obese in adulthood [33]. Combined, these factors are associated with an increased risk of premature mortality and may contribute to poor learning outcomes [34, 35]. This is partially attributable to higher school absences, repeating grades, lower engagement in class, dropping out of school, social isolation due to bullying, and reduced physical activity [29, 36].

The ability of health systems in developing countries to address population needs is being jeopardized by the extensive burden of NCDs [37]. The economic burden of obesity is felt at the individual, household, and national levels. This is due to increased healthcare expenditure, reduced productivity, absenteeism from work, disability, and mortality [38, 39]. If the status quo remains, the economic impact of overweight and obesity is predicted to continue rising, with the most significant burden concentrated in LMICs [40]. Even more concerning, in most LMICs, overnutrition coexists with undernutrition, which further strains the health systems [41–45].

We found that children from private schools had significantly higher odds of being overnourished. This agrees with the findings of a scoping systematic review examining the factors contributing to the risk and health conditions linked to childhood obesity in SSA, which showed significantly higher rates of overnutrition in private schools compared to public ones [1]. This is like the findings of a study conducted in Ethiopia among 736 children, which also found a higher prevalence of overweight and obesity among children in private schools compared to government schools [46]. The observed phenomenon might be explained by the fact that affording private schools is a proxy indicator of the higher socioeconomic status of the children's families. Additionally, children in private schools accrue higher sedentary time due to motorized transport, fewer PE lessons, and access to electronics used for entertainment and indoor gaming, such as televisions and high-energy foods and drinks [47–49].

Despite PE being a mandated lesson in the Kenyan curriculum for primary education, 13.5% of the children reported having yet to participate in a PE class in the week preceding the survey. This finding agrees with the findings of the National Assessment System for Monitoring Learner Achievement (NASMLA) Class 3 study. In this study, the Kenya National Examinations Council (KNEC) reported that in 2018, about 18.8% of pupils did not attend P.E. lessons. A similar study in Kenya among class seven pupils revealed that nearly half (47.9%) did not participate in PE lessons (KNEC, 2020). An even higher proportion, 32.1%, did not attend PE classes in South Africa [28]. This could indicate ineffective delivery and a lack of emphasis on the importance of PE. Several studies have documented barriers to PE delivery, including inadequate training, negative perceptions, lack of expertise, and a lower burden of accountability on the part of the teachers. Furthermore, inadequate resources and lack of support from colleagues affect the quality of these programs [50–52].

Globally, physical inactivity has been rising across all population groups partly due to inactivity during leisure time and increased sedentary behavior [27]. We found a mean PA score of 2.85, nearly half of the respondents were classified as inactive, with a PA score of < 2.74. This finding is similar to findings from several studies demonstrating a decrease in PA in school children. This decrease has been linked to deficiencies in curriculum materials, culture, gender, time, and built environment constructs, such as inadequate play facilities and infrastructure, motorized school transport, and street connectivity [53–55]. Our study, therefore, emphasizes the need to promote increased physical activity among children. Adequate physical activity in children provides various health benefits during growth and later in life. For children and adolescents, an average of 1 h of MVPA daily is associated with improved bone health, cardiorespiratory and muscular health and fitness, cognition, reduced adiposity, and decreased risk of depression [18, 19]. Our results showed no significant association between the average physical activity levels and gender. This contrasts with the findings of a study conducted in South Africa, which concluded that the mean physical activity for boys was significantly higher than that for girls [53]. Another study conducted in Senegal found a significant difference in objectively measured physical activity in the pupils [56].

We found no significant association between physical inactivity and overnutrition. This result is counterintuitive, especially because physical inactivity increases energy expenditure, decreasing the risk of excessive weight gain. Through the years, findings assessing the relationship between physical activity and overnutrition have been contradictory, further pointing to the multifactorial nature of overnutrition occurrence [57, 58]. Additionally, the lack of a significant association between walking to school and overnutrition in the adjusted model stratified by school type concurs with previous evidence positioning socioeconomic status center stage as a predictor. Previous research has consistently shown that children who attend private schools, often indicative of higher socioeconomic status, have higher rates of overnutrition. This predicted outcome underscores the importance of interventions that go beyond individual behaviors to address broader structural determinants of health. It demonstrates the intricate dynamic of socioeconomic status, built environment, and obesity risk [47–49]. The nonsignificant observation in this study could also be attributed to residual confounding by unmeasured factors, such as family eating habits or security in the neighborhoods, which may also reduce the effect of walking [59].

Our study has some limitations, most notably its cross-sectional nature, which prevents making assertions about causation and effect. Second, the use of BMI for age, although recommended due to its practicality in epidemiologic studies, has shown to have reliability limitations in estimating nutritional status. Third, this study did not evaluate the impact of parental/family and community-level factors, puberty, health, and genetic factors on obesity, which is a significant drawback. Furthermore, while the questions were specifically tailored to be suitable for this age group, we cannot dismiss the potential for recall bias. The participants were instructed to recall incidents that took place within the past week.

The limitations notwithstanding the results provide useful evidence of the growing prevalence of overnutrition and physical inactivity among school children. The findings could be used to inform the development of comprehensive school health programs emphasizing adequate physical activity. This could be made possible through the provision of adequate resources and an adequate teacher-to-pupil ratio. Community engagement can also be implemented to inspire caregivers to discourage sedentary practice at home and instead encourage both indoor and outdoor play. Additionally, at the national level, physical activity guidelines and curricula should be implemented to ensure healthy physical activity among school-going children.

## Figures and Tables

**Table 1:** Characteristics of the school children in Thika Town, Kenya.

Characteristic	*n* (%)
School type	
Public	146 (52)
Private	135 (48)
Age in years	
8–9	95 (33.8)
10–11	186 (66.2)
Gender	
Female	155 (55.2)
Male	126 (44.8)
Grade	
Grade 4	145 (51.6)
Grade 5	125 (44.5)
Grade 6	11 (3.9)
BMI for age Z-scores	
<−2 SD (underweight)	63 (22.4)
>−2 SD to < 1 SD (normal)	187 (66.6)
>+1SD (overweight)	29 (10.3)
>+2 SD (obese)	2 (0.7)

**Table 2:** Physical activity level of school children in Thika Town, Kenya (*N = *281).

Characteristic	Number of days	*n*	%
No. of days school children participated in physical education classes in a week	0	38	13.5
	1	105	37.4
	2	45	16
	3	43	15.3
	4	14	5
	5	36	12.8
	Total	281	100

Time spent in each PE session	Duration in minutes	*n*	%
	≤ 35 min	194	79.8
	≥ 36 min	49	20.2
	Total	243	100

Routinely walking from home to school	Response	*n*	%
	Walks to school	184	65.5
	Does not walk to school	97	34.5
	Total	281	100

Duration of walks by children who routinely walked to school	Duration in minutes	*n*	%
	≤ 15 min	85	46.2
	16–30 min	28	15.2
	≥ 31 min	71	38.6
	Total	184	100

Average physical activity score per the PAQ-C	Average score	*n*	%
	1	3	1.1
	2	80	28.5
	3	153	54.4
	4	44	15.7
	5	1	0.4
	Total	184	100

Classification of physical activity	Classification	*n*	100
	Physically active	154	54.8
	Physically inactive	127	45.2
	Total	281	100

**Table 3:** Relationship between the children's characteristics and overnutrition in school children in Thika Town, Kenya (bivariate).

Variable	Not overweight	Overweight	*χ* ^2^	*p* value
School type			9.050	**0.003**
Public	122 (84)	24 (16)		
Private	128 (95)	7 (5)		
Age in years			0.037	0.847
8–9	85 (89.5)	10 (10.5)		
10–11	165 (88.7)	21 (11.3)		
Gender			0.845	0.358
Female	135 (87)	20 (13)		
Male	115 (91)	11 (9)		
Grade^¥^			2.939	0.214
Grade 5	108 (86)	17 (14)		
Grade 4	133 (92)	12 (8)		
Grade 6	9 (82)	2 (18)		
Participating in PE^¥^			0.202	0.653
Participated in PE	217 (89)	26 (11)		
Did not participate in PE	33 (87)	5 (13)		
Routinely walks from home to school			7.415	**0.006**
Walks to school	171 (68.4)	13 (41.9)		
Does not walk to school	79 (31.6)	18 (58.1)		
Duration of walks by children who routinely walked to school^¥^			3.723	0.155
≤ 15 min	82	3		
16–30 min	69	7		
≥ 31 min	20	3		
Physical activity score^¥^			0.923	0.407
1	2 (67)	1 (33)		
2	73 (91)	7 (9)		
3	136 (89)	17 (11)		
4	39 (89)	5 (11)		
5	0 (0)	1 (100)		
Level of physical activity			0.592	0.442
Physically active	135 (88)	19 (12)		
Physically inactive	115 (91)	12 (9)		

*Note:* The bold values denote a statistically significant association between the variables at the *p* < 0.05 level.

^¥^Fisher's exact test.

**Table 4:** Multivariate analysis of factors associated with overnutrition in school children in Thika Town, Kenya.

Variable	cOR (95% C.I)	*p* value	aOR (95% C.I)	*p* value
School type				
Public (ref)	1		1	
Private	3.597 (1.496–8.652)	**0.005**	2.641 (1.013–6.887)	**0.0047**
Routinely walks from home to school				
Walks to school (ref)	1		1	
Does not walk to school	2.997 (1.399–6.419)	**0.004**	2.017 (0.875–4.650)	0.1

*Note:* The bold values denote a statistically significant association between the variables at the *p* < 0.05 level.

## Data Availability

The data that support the findings of this study are available on request from the corresponding author. The data are not publicly available due to privacy or ethical restrictions.
